# Nurses and the Ministry of Health

**Published:** 1919-11-01

**Authors:** 


**The Editor's Letter-Box——(*continued*).**

### NURSES AND THE MINISTRY OF HEALTH.

*To the Editor of* The Hospital.

Sir,—It is evident "Ierne" holds a brief for the Ministry of Health; but, even so, it is advisable to look at facts squarely from the standpoint of the nursing services.

The Ministry of Health has drawn up a special curriculum for students desirous to become health visitors, the period of training extends over two years; but, taking into consideration the theoretical and practical knowledge obtained by a certificated nurse who has successfully passed through a recognised training-school, the Ministry is willing to remit one year's study in her favour. Briefly, the Ministry of Health states, and states clearly, that the experience and efficiency obtained in the training-schools of Great Britain are equivalent to the standard of knowledge imbibed by a student during a year's study in one of the women's colleges.

Has "Ierne" considered what would be the attitude of the B.M.A. if the Ministry of Health proposed to appoint, as officers to the welfare centres, on equal terms with their qualified professional brethren, men or women who had taken an abridged course in the science of infant welfare?

We are also reminded that the Ministry of Health has referred to the scandalous manner in which nurses are underpaid. "Ierne" laments the fact that no organised efforts have been made by any school of nurses' leaders to remedy this evil, but he fails to note that until the College of Nursing was incorporated there were no recognised leaders sufficiently influential to demand or to receive a hearing.

Also, can "Ierne" explain satisfactorily the reason why the Ministry of Health, if it is really perturbed as to the exploitation of the nursing services, does not take definite steps towards putting an end to what may be considered a national scandal?

We are still awaiting the Bill for the State Registration of Nurses which the Government promised to introduce through the medium of the Ministry of Health. No thinking individual can regard State registration as "a panacea for all ills," but it can, and will, define the status of the trained certificated nurse, and strengthen the hands of those men and women who are endeavouring to stamp the nursing services indelibly as a body of professional women.

Finally, is "Ierne" aware that whilst we are patiently waiting to receive "the crumbs which may, or may not, fall from the rich man's table," the Cottage Benefit Nursing Association are advertising in the daily papers for women who, on completion of one year's training, are assigned the status of a nurse, and are expected to combine in their work the duties of a professional woman and a home help, to the detriment of both? If "Ierne" will assimilate and mentally digest these facts he will realise that in the minds of many members of the nursing services the present attitude of the Ministry of Health has aroused suspicion and distrust.—Yours faithfully,

